# Proactive huddles to reduce missed nursing care; the mediating roles of personal situational awareness and rational coordination: A cluster randomized pre post intervention study

**DOI:** 10.1016/j.ijnsa.2025.100448

**Published:** 2025-11-10

**Authors:** Marina Vexler, Anat Drach-Zahavy, Einav Srulovici

**Affiliations:** a The Cheryl Spencer Department of Nursing, University of Haifa, Haifa, Israel; b Meir Medical Center, Clalit Health Services, Israel

**Keywords:** Proactive huddles, intervention, missed nursing care, nurse, relational coordination, situational awareness

## Abstract

**Background:**

Missed nursing care, defined as the failure to deliver essential patient care, has adverse effects on patients, nurses, and healthcare organizations. While efforts to reduce missed care exist, few interventions have been fully evaluated, and the mechanisms through which these interventions work remain poorly understood.

**Objectives:**

This study aimed to develop, implement, and evaluate proactive huddles as a process to reduce missed nursing care in hospital inpatient wards. Additionally, the study examined the mediating role of personal situational awareness (cognitive mechanism) and relational coordination (motivational mechanism) in the relationship between proactive huddles and missed care.

**Design:**

A cluster-randomized pre–post intervention design

**Methods:**

Data were collected from March 2022 to May 2023 from six internal and four surgical wards in a medium-sized hospital. Wards were randomized into intervention (n = 85) and control (n = 95) groups. Nurses in the intervention group participated in daily huddles over three months, while those in the control group continued with standard care practices. The MISSCARE survey, Relational Coordination Survey, Situational Nursing Awareness Probe – Missed Nursing Care Edition (SANP-MNC), National Aeronautics and Space Administration (NASA) Task Load Index, and sociodemographic characteristics were assessed pre- and post-intervention. Mediation models were analyzed using mixed-linear model analyses.

**Results:**

The proactive huddle intervention significantly reduced missed nursing care (β =0.123, p< 0.001), with partial mediation observed through improved relational coordination (β =-0.125, p< 0.001). However, while the intervention increased personal situational awareness (β =-0.142, p< 0.001), this cognitive mechanism did not mediate the relationship between the intervention and missed care.

**Conclusions:**

Proactive huddles were effective in reducing missed nursing care by improving team communication and collaboration. Although situational awareness increased, the high workload and limited resources may have hindered nurses' ability to act on situational awareness. For proactive huddles to maximize their potential, additional support systems are needed to enable nurses to address care challenges effectively.

**What is already known about the topic?**

 

• Missed nursing care remains a persistent challenge in healthcare despite ongoing interventions and research.
• Randomized controlled study designs aimed at reducing missed nursing care are limited.
• Proactive huddles have shown benefits for nurses in multidisciplinary teams, particularly in improving communication and teamwork.

**What this paper adds?**

 

• This is the first randomized controlled study design that demonstrates the effectiveness of proactive huddles in reducing missed nursing care.
• Relational coordination was identified as a key mediating mechanism, explaining how proactive huddles contribute to a reduction in missed care.
• Although proactive huddles enhanced nurses' situational awareness, this improvement did not translate into a decrease in missed nursing care.

Alt-text: Unlabelled box

## 1 Introduction

Missed nursing care — any required patient care task that is delayed or omitted, either partially or fully (Kalisch et al., 2009) — is a significant barrier to achieving patient safety and quality of care. Despite being fundamental to nursing practice, ensuring patient safety and quality care is challenging, especially in hospital settings (Amrolahi-Mishavan et al., 2022). Evidence shows that failure to deliver necessary nursing care is common (Imam et al., 2023), and the prevalence of missed nursing care is worsened by negative organizational factors such as unsupportive environments, high nurse workloads, and high patient-to-nurse ratios (Bayadsi et al., 2024; Cho & Steege, 2021; Crincoli et al., 2022). Additionally, nurse-specific factors like fatigue (Cho & Steege, 2021), insufficient knowledge, low work effectiveness (Peng et al., 2023), and high cognitive load (Kerari & Innab, 2021) further contribute to missed nursing care. The consequences of missed nursing care affect patients, nurses, and healthcare organizations, compromising care quality and patient outcomes (Chaboyer et al., 2021).

Despite strategic initiatives by organizations to reduce missed nursing care, the issue persists, underscoring the need for targeted interventions that address its root causes. The organizational and nurse-specific factors triggering missed nursing care indicate that interventions need to target both teamwork and individual cognitive processes to effectively address the phenomenon. Yet, only scant research has examined the effectiveness of targeted interventions to reduce missed nursing care (Schubert et al., 2021), and these often overlooked the underlying mechanisms through which these interventions reduce missed nursing care. This paper seeks to address these gaps by developing, implementing, and evaluating the effectiveness of a proactive huddle intervention—brief, structured meetings where healthcare team members discuss patient care, share critical information, and coordinate their actions as means to improve patients’ quality and safe care (Edbrooke-Childs et al., 2018; Murphy, 2023). It aims to explore two underlying mediating mechanisms: a motivational mechanism focused on enhancing relational coordination (i.e., the quality of communication, shared goals, and mutual respect among team members; Gittell, 2002); and a cognitive mechanism aimed at improving situational awareness (i.e., nurses’ ability to perceive, interpret, and anticipate patient care situations; Endsley, 1995). The motivational mechanism suggests that proactive huddles reduce missed nursing care by fostering cooperation and coordination among nurses (Aldawood et al., 2020). Meanwhile, the cognitive mechanism proposes that huddles reduce missed nursing care by enhancing nurses' situational awareness (Cosgrove et al., 2023). Moreover, this paper aims to address methodological issues of previous intervention studies to reduce missed nursing care (Schubert et al., 2021) by employing an RCT methodology, serving as a golden standard for intervention research to ensure the accuracy and reliability of the findings (Steeger et al., 2021).

## 2 Background

### 2.1 Missed Nursing Care

Growing evidence suggests that missed nursing care constitutes a widespread global problem with detrimental effects on patients’ safety in many settings, including hospitals (Ball et al., 2016) and community settings (Phelan et al., 2018). It affects patients across the lifespan, including neonates and children (Bagnasco et al., 2019; Tubbs-Cooley et al., 2015) to adults in hospital settings (Juvé‐Udina et al., 2020). Research to date has primarily focused on the tasks most prone to being missed, as well as the structural and organizational factors leading to missed nursing care and its outcomes for patients, nurses, and organizations (Chaboyer et al., 2021; McCauley et al., 2020;). The nursing tasks most frequently missed include patient discharge planning and teaching, turning patients every 2 hours, full documentation of all necessary data, and patient assessments performed each shift (Abere et al., 2024). Furthermore, the emotional load hospital nurses typically face in their daily life (Sitterding et al., 2012; Yekita et al., 2024) may lead nurses to forget important information or tasks, affecting their performance (Kerari & Innab, 2021).

Nine systematic reviews have identified factors contributing to missed nursing care, such as poor management, competing priorities in nurses' workflows, inadequate staffing levels, improper load distribution, insufficient staff experience, poor teamwork, lack of communication during shift changes, and the demeanor and attitude of staff members (Papathanasiou et al., 2024). Additionally, studies show that missed nursing care could result in increased mortality, adverse events and failure to rescue (Willis & Brady, 2022). These findings led to development and implementation of interventions to decrease missed nursing care.

### 2.2 Interventions to reduce missed nursing care

Research so far has explored the effectiveness of several interventions to decrease missed nursing care. These strategies aim to boost patient safety and elevate the overall standard of care delivered (Longhini et al., 2021). Generally, interventions were divided to two main strategies: process improvement (e.g., improving nurses' teamwork, reminders) and structural improvement (e.g., sufficient resources, appropriate work environment). A systematic analysis performed by Schubert and colleagues (2021), addressed the effectiveness of 11 different interventions to reduce missed nursing care. Eight of them were process interventions and three were structural. Process interventions include integrating of care reminders (e.g. Lytle et al., 2015) and optimization of nursing care process that focused on a specific task **(**e.g. Gittner et al., 2015). Structural interventions involve improving nurses’ qualifications (e.g. Kalisch et al., 2013) and increase in nurse staffing levels (e.g. Cho et al., 2015). Although the authors found the interventions to be promising, they also voiced some concerns. First, the theoretical and empirical literature about specific mechanisms through which these interventions lead to reduced missed nursing care is unclear. Second, theses interventions might be too limited in scope, addressing specific tasks such as medication administration, rather than tackling the broader issue of missed nursing care. The authors also highlighted several methodological issues, including low participation rates and high costs. While reminders proved to be an effective and low-cost method for reducing missed nursing care, the medium-low quality of the studies prevented clear conclusions. Thus, identifying the best method to prevent missed nursing care remains a challenging task.

In the last decade, the implementation of regular proactive huddles—brief, timely meetings among staff aimed at enhancing quality of care (Pimentel et al., 2021; McGilton et al., 2023)— has brought significant changes to the work culture of nursing teams (McGilton et al., 2023). This method provides an essential platform for nursing professionals to engage in open dialogue, fostering an environment where topics such as current workloads, upcoming tasks, patient care plans, priorities, and potential challenges are openly discussed (Pimentel et al., 2021). It offers opportunities for nursing teams to address issues in real-time, coordinate patient care plans, and enhance situational awareness and relational coordination, which are critical to preventing missed nursing care as part of a broader commitment to patient safety (Murphy et al., 2023).

### 2.3 The research model and hypotheses

**Fig. 1:**
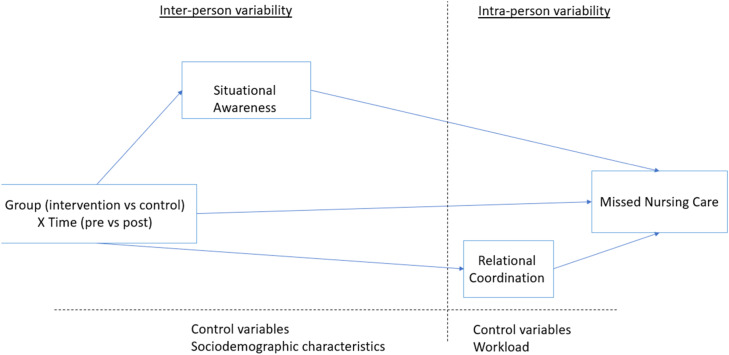
Conceptual framework.

To address gaps in the literature, we develop and test a research model suggesting that proactive huddles reduce missed nursing care through two parallel mechanisms: cognitive and motivational. The cognitive mechanism posits that proactive huddles increase nurses' available cognitive resources by enhancing their situational awareness, enabling them to complete their tasks more effectively (Endsley, 1995). Meanwhile, the motivational mechanism suggests that proactive huddles reduce missed nursing care by fostering the development of nurses' relational coordination (Gittell, 2002; see Fig. 1). To capture these two mechanisms, the current research is grounded in two main theories: missed nursing care theory (Kalisch et al., 2009), which suggests that missed nursing care is largely a result of nurse overload and insufficient available resources, and resource allocation model (Kanfer & Ackerman, 1989), which emphasizes that individual performance critically depends on the availability of cognitive resources.

### 2.4 The cognitive mechanism

The cognitive mechanism proposes that proactive huddles decrease missed nursing care by enhancing nurses’ situational awareness. Situation awareness—the cognitive ability to gather information from the environment, recognize and understand it, and anticipate its implications for future actions— is considered a vital non-technical skill for nurses (Endsley, 1995 Allard, et al., 2020). Positioned at the patient’s bedside, nurses are the frontline safeguard ensuring patient care quality and safety (Cosgrove et al., 2023). In high-pressure environments like hospitals, nurses frequently encounter complex and rapidly changing situations that can lead to cognitive overload (Endsley, 2012). Proactive huddles play a critical role in alleviating cognitive burden by offering structured opportunities for brief breaks from routine work. These short time-outs enable nurses to process real-time information and prioritize tasks, focusing on the most urgent aspects of patient care. The resulting cognitive clarity enhances nurses' situational awareness, improving their ability to anticipate and address patient needs, thereby reducing instances of missed nursing care (Ghaderi et al., 2022). These breaks are important as environmental factors (e.g., noise, equipment issues, time pressure), individual factors (e.g., anxiety, fatigue, stress), and cognitive factors (e.g., limited attentional capacity, information overload, task interruptions) commonly present in nursing workplaces can impair situational awareness (Endsley, 2012; Wang et al., 2021).

Our model, which suggests that proactive huddles may increase situational awareness, relies on substantial research demonstrating the multifaceted benefits of huddles in augmenting healthcare professionals' personal situational awareness (Cosgrove et.al., 2023; Lamphere et al., 2023) and fostering a proactive and safety-oriented culture within healthcare organizations (Chapman et al., 2020). The real-time discussions during huddles appear to provide nurses with a heightened understanding of the current clinical context and potential challenges to achieving high-quality patient care, as well as a greater ability to develop strategies to overcome these challenges (Pimentel et al., 2021). Although research on the direct relationship between situational awareness and missed nursing care is limited, evidence suggests that participating in huddles may decrease missed nursing care by enhancing nurses’ situational awareness.

### 2.5 The motivational mechanism

The motivational mechanism proposes that proactive huddles reduce missed nursing care by fostering relational coordination (RC) – a framework emphasizing communication, shared goals, and mutual respect among team members (Gittell, 2002). In complex and high-pressure environments like healthcare, effective coordination of patient care activities relies heavily on strong interpersonal relationships and collaboration. Proactive huddles create structured opportunities for team members to clarify roles, exchange insights, and synchronize their efforts, all of which are crucial for preventing errors and ensuring comprehensive care (Gittell, 2002, 2011; Havens et al., 2010). By facilitating open communication and shared responsibility, these huddles foster a collective commitment to patient outcomes, leading to more effective teamwork and fewer instances of missed nursing care (Burr et al., 2021; Pimentel et al., 2021).

Research consistently supports the positive impact of huddles on team coordination and communication. A recent scoping review found that 67% of studies demonstrated that huddles improved collaboration and communication across clinical roles (Lai et al., 2023; Pimentel et al., 2021). While existing research predominantly highlights huddles' benefits for communication, teamwork, and patient safety, there has been limited investigation into their direct role in addressing missed nursing care. Huddles are typically used to review patient care plans, manage workloads, and address safety concerns (Burr et al., 2021; Lin et al., 2022), but their potential as a platform to tackle missed nursing care remains underexplored. Nevertheless, evidence from studies on missed care indicates that improving relational coordination is a proven strategy for reducing missed nursing care, as high-quality communication and strong professional relationships are critical for ensuring that essential nursing tasks are completed (Chaboyer et al., 2021). By fostering these key elements, proactive huddles enhance team dynamics and contribute to a more resilient and coordinated approach to patient care, addressing the pervasive issue of missed nursing care in healthcare settings (Gittell, 2002).

The following hypotheses are derived from the theoretical and empirical foundations outlined:

Hypothesis 1: Nurses who participated in the proactive huddle intervention will report lower levels of missed nursing care compared to those in the control group.

Hypothesis 2: The interaction effect of group (intervention vs. control) and time (pre-intervention vs. post-intervention) on reducing missed nursing care will be mediated by personal situational awareness.

Hypothesis 3: The interaction effect of group (intervention vs. control) and time (pre-intervention vs. post-intervention) on reducing missed nursing care will be mediated by relational coordination.

## 3 Method

### 3.1 Design

This study employed a cluster-randomized pre–post intervention design, with randomization at the level of nursing wards (clusters). The intervention was implemented over a three-month period, with data collected at two separate time points, before and after the intervention. The study was conducted in a medium-sized hospital affiliated with a university, serving both rural and urban communities. The intervention was conducted in ten nursing wards providing direct patient care, including six internal medicine wards and four surgical wards, which were deemed eligible for participation. The inclusion criteria involved internal and surgical wards with similar inpatient profiles and workloads. The exclusion criteria included wards with unique specialization, such as emergency rooms and intensive care units, as well as relatively small wards like otolaryngology and ophthalmologic. Data were collected from individual nurses nested within these ward clusters. Wards (not individual nurses) were randomized in a 1:1 ratio, with five wards assigned to the intervention group and five to the control group. Due to the nature of the intervention, blinding the researcher to the group assignments was not feasible.

### 3.2 Sample

**Fig. 2:**
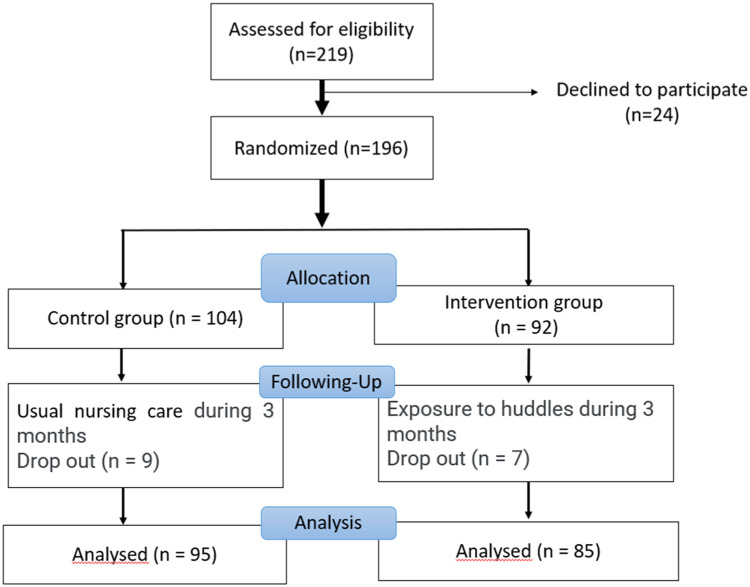
Participant flow.

In total, 219 nurses were screened for eligibility. The inclusion criteria included all nurses working regularly in the participating wards. Nurses who were excluded from the study included certified nursing assistants, non-permanent nurses, nurse students, nurse educators, nurses working only night shifts, and head nurses in the ward. Among the 219 nurses, 196 (89%) agreed to participate and were divided into the intervention group (n =92) and the control group (n=104). A total of 92% of nurses (n=85) in the intervention group and 91% of nurses (n=95) in the control group completed the study (Fig. 2). Some nurses chose not to participate due to time constraints or lack of interest.

Sample size was computed for linear mixed models to detect an effect size of 0.30 with power = .95, assuming ICC = .15 and 5 observations per nurse, yielding a recommended nurse-level sample of 180 (≈953 observations). The study exceeded this target (180 nurses; 6 observations per nurse; 1,080 observations). Because randomization occurred at the ward level but outcomes were repeatedly measured at the nurse level, the analytic plan focused on repeated measures nested within nurses; the ward-level assignment is represented by the fixed “Group” factor (intervention vs. control)**.** This approach provides adequate statistical power while addressing the clustered structure of the data within the linear mixed model framework.

### 3.3 The intervention

The proactive huddle intervention was developed and validated in a pilot study, demonstrating good fidelity to the intervention (for details please see Vexler et al., 2025a). Briefly, it was structured around several key principles. First, the huddle was a short, organized meeting specifically focused on identifying and addressing missed nursing care. These meetings were scheduled at a designated time and place within each ward. The environment of the huddles was supportive, non-judgmental, and non-threatening, to encourage open communication among the nursing staff. The meetings followed a standardized agenda and script that included safety-related prompts, such as inquiries about unusual events (e.g., resuscitations, patient falls, family incidents). At the end of each session, the huddle leader completed a summary document to record the discussion (supplementary material 1).

The huddles were conducted daily at consistent times during both morning and evening shifts, excluding weekends. In the morning shift, huddles took place at 11:30 a.m., and during the evening shift, they occurred at 7:00 p.m. Each huddle was led by the charge nurse and lasted up to 15 minutes, though the average duration was approximately five minutes. All nurses on the shift participated, typically about four nurses per ward. Guided by a huddle script covering critical safety tasks such as medication administration, repeated assessments, and wound care (supplementary material 2), each nurse provided a verbal update on their current tasks, any anticipated delays or incomplete tasks, factors impacting their workload, and requests for assistance. The nurses then discussed potential solutions to prevent missed care, and the charge nurse concluded the session by summarizing these suggestions and delegating tasks to resolve any issues. For instance, if one nurse was unable to complete a task, such as applying a pressure dressing, due to another obligation, another nurse would offer to handle it. In another scenario, a nurse might forget to request a physician to insert an IV cannula, leading to a delay in medication administration. The huddle provided an opportunity for the nurse to recall this oversight and resolve the issue promptly.

## 4 Procedure

The study was divided into three phases:

**Phase1: Pre-intervention.** Conducted in ten wards between July and October 2022, this phase involved collecting baseline data. Nurses were asked to complete a set of questionnaires to measure relational coordination and sociodemographic variables. In addition, nurses assessed the frequency of missed nursing care, their personal situational awareness, and their workload at the end of three different shifts.

**Phase 2: Intervention.** The proactive huddle intervention, described above, was implemented in five randomly assigned wards between November 2022 and January 2023. The remaining five wards, assigned to the control group, continued to provide care as usual. Prior to the start of the intervention, participants underwent a training session on the purpose of the huddles, the process, and the rules guiding their participation.

**Phase 3: Post-intervention.** This phase took place between January and May 2023 and followed the same data collection process as Phase 2. Nurses from all ten wards were asked to complete the relational coordination questionnaire once again and to assess the frequency of missed nursing care, their personal situational awareness, and their workload at the end of three shifts, as they had done during the pre-intervention phase.

While joining the study was voluntary, the intervention was integrated into ward practices, requiring all nurses in the intervention wards to participate in proactive huddles as part of their daily workflow. Nurses who chose not to participate or later withdrew from the study were not included in the survey assessments, but they continued to be exposed to the intervention to maintain its implementation within the ward team.

## 5 Measures

### 5.1 Outcome variable - Missed Nursing Care

*Missed nursing care* was assessed using the 22‐item MISSCARE survey (Kalisch & Williams, 2009), which allows nurses to self‐assess missed nursing care during their last shift on a 4‐point Likert scale (1 = rarely missed; 4= always missed; 9= not relevant). An example item: “Full documentation of all necessary data”. The average level of missed nursing care for each nurse was calculated based on their responses to all 22 items. The reliability of the scale was found to be excellent, with values across three different shifts before the intervention being 0.90, 0.92, and 0.93, and after the intervention, the values were 0.93, 0.92, and 0.93.

### 5.2 Independent variables – Group and Time

*Group assignment* was determined based on whether wards were assigned to the intervention or control conditions. Nurses in the intervention group participated in the proactive huddle intervention, while those in the control group continued with standard nursing practices. The intervention group was coded as 1, and the control group as 0.

*Time* was captured based on whether the data were collected before or after the intervention was implemented. Pre-intervention data were collected prior to the initiation of the proactive huddle intervention, and post-intervention data were collected after the completion of the three-month intervention period. The pre-intervention period was coded as 0, and the post-intervention period was coded as 1.

### 5.3 Mediating variables – Relational Coordination and Situational Awareness

*Relational coordination* was measured using a 7-item survey (Gittell, 2002). Nurses rated each item on a 7-point Likert-type scale, ranging from 1 (never) to 7 (always), to assess the frequency and quality of communication and relationships among the nursing staff in their ward. Example items included: “How often do you communicate with the nursing staff in your ward regarding the patient's condition?” and “How much do people on your ward's nursing staff respect you and your mission to improve the quality of patient care?”. Cronbach’s alpha for this scale at baseline was 0.75, and after the intervention, it was 0.84.

*Personal situational awareness* was measured using the Situational Nursing Awareness Probe – Missed Nursing Care Edition (SNAP-MNC) questionnaire, developed and validated by Vexler et al., (2025b). Nurses responded to five open-ended questions, referring to the three components of situational awareness, as defined by Endsley, (1995). Participants responded qualitatively to five open-ended questions. Item 1 assessed nurses’ perceptions of the reasons for missed nursing care (MNC); their responses were compared to a validated, evidence-based list of 20 missed nursing care factors, and a ratio score was calculated based on the number of accurately identified factors. For Items 2 through 5, nurses’ responses were evaluated using a 3-point scale (0 = no comprehension/projection, 0.5 = partial, 1 = full). The overall situational awareness score was computed as the average of all item scores, ranging from 0 to 1, with higher scores indicating greater situational awareness. Example items included “The cause of delaying or missing nursing care” and “How do you think it affected nursing care performance and/or completion?” . Structural validity demonstrated support for a uni-scale structure. The questionnaire demonstrated excellent interrater validity (Kappa=.94) and Cronbach’s were above .88 (Vexler et al., 2025b).

### 5.4 Control variables

*Workload* was measured using the NASA Task Load Index (NASA-TLX) (Hart, 2006), which assesses subjective workload. The scale examines six subscales: Mental Demand, Physical Demand, Temporal Demand, Performance, Effort, and Frustration. Nurses reported their workload on a 20-point scale (1 = "low load" to 20 = "high load") for each subscale. Each nurse completed the index six times: three times before the intervention and three times after. In this study, Cronbach's alpha for workload was 0.84, 0.85, and 0.84 at baseline and 0.85, 0.83, and 0.84 after the intervention.

*Nurse characteristics* were measured, including gender (male/female) and nurse seniority (years since graduation), as these factors have been previously linked to missed nursing care (Jones et al., 2015).

### 5.5 Analysis

Statistical analyses were conducted using IBM SPSS (version 27) (IBM SPSS, Chicago, IL) and R (version 4.1.0). The socio-demographic characteristics of the nurses in each group were compared using the t-tests and chi-squared tests. Paired-sample t-tests were conducted in R to examine within-group changes (pretest vs. posttest) for each outcome variable in the intervention and control groups. The emmeans package was used to compute estimated marginal means and pairwise comparisons. A significance level of α = .05 was applied to all analyses. To account for the nested data structure, with repeated measures collected for each nurse, linear mixed-effects models (LMMs) were used to analyze the outcome variables. An intention-to-treat analysis was employed to assess the effectiveness of the intervention. These models included time, group, and their interaction as fixed effects, with random intercepts for each nurse to account for within-subject variability. Following Baron and Kenny's (1986) mediation procedure, two models were run for each hypothesized mechanism (personal situational awareness or relational coordination).

In Model 1, the mediator (either situational awareness or relational coordination) was treated as the outcome variable. This model included three steps: (1) inclusion of control variables; (2) inclusion of explanatory variables (time and group); and (3) testing the interaction between time and group. A significant interaction term indicated moderation.

In Model 2, missed nursing care was treated as the outcome variable. This model included the first three steps of Model 1, followed by (4) testing the direct effect of the mediator (situational awareness or relational coordination) on missed nursing care, and (5) testing for a mediating effect by including both the interaction (group*time) and the mediator in the model. A non-significant interaction term, along with a significant mediator effect, supported the presence of mediation.

### 5.6 Ethical consideration

Ethics approval was obtained from the university ethics committee (Approval No. 088/22) and the hospital’s Institutional Review Board (Approval No. 0012-22-COM1) prior to the commencement of the study. All participating nurses provided informed consent by signing a consent form, which clearly outlined their voluntary participation. Participants were informed that they could withdraw from the study at any point without facing any repercussions or consequences to their professional standing.

## 6 Results

### 6.1 Socio-demographic data

Table 1 shows the socio-demographic and occupational characteristics of the total sample, stratified by the intervention and control groups. The mean age of participants was 39.36 years (SD = 10.61), and 75% were women. Most nurses held a Bachelor's degree (77.2%), while a smaller percentage had a Master's degree (5.6%). The only significant difference between the groups was in years as nurses and years of experience in the ward, with nurses in the intervention group having more experience than those in the control group (p < 0.001). No missing data were reported for any outcome measures.

**Table 1:** Comparison of demographic variables of all participants (N = 180), within and between intervention group (n = 85) and control group (n = 95).

Demographic characteristics	Total (n=180)		Intervention (n = 85)		Control (n = 95)		P-value
	N	%	N	%	N	%	
Female	135	75	66	77.6	69	72.6	0.438
Male	45	25	19	22.4	26	27.4	
Age in years, mean (SD) [min-max]	39.36 (10.61) [23-64]		39.59 (11.16) [25-64]		39.16 (10.14) [23-64]		0.193
Education level:							0.195
Licensed practical nurse	7	3.9	5	5.9	2	2.1	
RN without Bachelor's degree	24	13.3	12	14.1	12	12.6	
RN with Bachelor's degree	139	77.2	66	77.6	73	76.8	
Master's degree	10	5.6	2	2.4	8	8.4	
Years as nurses mean (SD) [min-max]	10.6 (10.97) [0-40]		12 (11.9) [0.1-40]		9.3 (9.9) [0- 40]		0.001
Nurse's experience (years) in the ward, mean (SD) [min-max]	9.09 (9.6) [0-40]		10.6 (10.72) [0.1-40]		7.7 (8.3) [0-40]		<0.001
Work unit:							0.739
Medical unit	55	30.6	58	68.2	76	70.5	
Surgical unit	125	69.4	27	31.8	28	29.5	

### 6.2 Effectiveness of the intervention on study variables

Table 2 presents the descriptive statistics and t-test results for the study variables in both the intervention and control groups, measured before and after the intervention. Missed nursing care significantly decreased in the intervention group from pre- to post-intervention (t(899) = 3.98, p < .001), while no significant change was observed in the control group (p = .245). Situational awareness improved significantly in both groups, with a greater improvement in the intervention group (t (899) = -16.0, p < .001) compared to the control group (t(899) = -6.03, p < .001). Relational coordination also showed a significant improvement in the intervention group (t(899) = -3.95, p < .001), whereas no significant change was observed in the control group (p = .268). Lastly, overload increased significantly in both the intervention group (t(899) = -2.45, p = .015) and the control group (t(899) = -4.95, p < .001).

**Table 2:** Descriptive statistics and t-test results for study variables.

Measures	Group	Pretest (M±SD)	Posttest (M±SD)	t-test (df)	p-value
Missed nursing care	Intervention	1.59 (0.37)	1.49 (0.40)	3.98 (899)	<.001
	Control	1.59 (0.47)	1.62 (0.53)	-1.16 (899)	0.245
Situation awareness	Intervention	0.10 (0.09)	0.31 (0.24)	-16.05 (899)	<.001
	Control	0.11 (0.12)	0.19 (0.15)	-6.03 (899)	<.001
Relation coordination	Intervention	5.14 (0.77)	5.34 (1.02)	-3.95 (899)	<.001
	Control	5.14 (0.84)	5.19 (0.85)	-1.11 (899)	0.268
Overload	Intervention	10.16 (3.21)	10.58 (3.24)	-2.45 (899)	0.015
	Control	10.45 (3.02)	11.25 (3.27)	-4.95 (899)	<.001

### 6.3 Intervention fidelity

A total of 604 huddles were conducted, with a compliance rate of 87% and a participation rate of 84% (for details, please see Vexler et al., 2025a).

### 6.4 Hypothesis testing

**Fig. 3:**
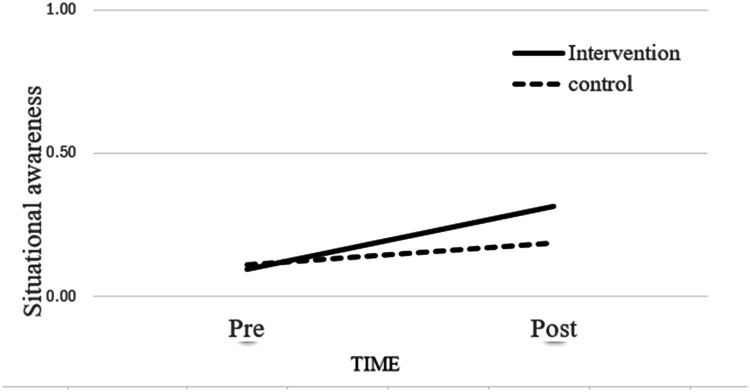
Effect of the interaction between time (pre vs. post) and group (intervention vs. control) on situational awareness.

Table 3 presents the findings for the cognitive mechanism, where situational awareness mediates the link between group*time and missed nursing care. In the results for situational awareness (Table 3, Model 1), the control variables—seniority, gender (male compared to female), and nurse’s subjective load—were not significantly related to situational awareness (Step 1). However, the direct effects of time (b = -.144, p < .001) and group (b =.058, p < .001) were significant (Step 2), as was the interaction effect between time and group (b = -.142, p < .001) (Step 3). The interaction effect depicted in Fig. 3 demonstrates that while both the intervention and control groups experienced improvements *in situ*ational awareness over time, the increase was significantly greater in the intervention group following the implementation of the huddle intervention, indicating a stronger impact of the intervention on situational awareness compared to usual care.

**Table 3:** Mixed Linear Model - Cognitive Mechanism.

	Model 1. Situational awareness as the dependent variable						
Variables	Step1. Control variables		Step 2. Explanatory variables		Step 3. Interaction effect		
	Estimate (SE)	p-value	Estimate (SE)	p-value	Estimate (SE)		p-value
Experience as a nurse	0.000 (0.001)	0.674	-0.001 (0.001)	0.305	-0.001 (0.001)		0.299
Gender (ref: female)	-0.024 (0.014)	0.076	-0.020 (0.013)	0.108	-0.021 (0.013)		0.103
Workload	0.003 (0.002)	0.106	0.001 (0.002)	0.504	0.002 (0.002)		0.348
Group (ref: control)			0.058 (0.011)	<0.001	0.129 (0.014)		<0.001
Time (ref: post-intervention)			-0.144 (0.010)	<0.001	-0.077 (0.013)		<0.001
Group (ref: intervention*post-intervention)					-0.142 (0.019)		<0.001
Δ-2 restricted log-likelihood	-612.654 (6)		-821.291 (8)		-870.437 (9)		
Residual	0.031 (0.031)	<0.001	0.025 (0.001)	<0.001	0.024 (0.001)	<0.001	
Variance	0.001 (0.001)	0.167	0.001 (0.001)	0.062	0.001 (0.001)	0.022	

	Model 2. Missed nursing care as the dependent variable									
Variable	Step 1. Control Variables		Step 2. Explanatory variables		Step 3. Interaction effect		Step 4. Mediation variable		Step 5. Mediation effect	
	Estimate (SE)	p-value	Estimate (SE)	p-value	Estimate (SE)	p-value	Estimate (SE)	p-value	Estimate (SE)	p-value
Experience as a nurse	0.005 (0.002)	0.043	0.005 (0.002)	0.034	0.005 (0.002)	0.033	0.005 (0.002)	0.045	0.005 (0.002)	0.033
Gender (ref: female)	0.245 (0.058)	<0.001	0.242 (0.058)	<0.001	0.243 (0.058)	<0.001	0.242 (0.058)	<0.001	0.243 (0.058)	<0.001
Workload	0.011 (0.004)	0.009	0.013 (0.004)	0.004	0.012 (0.004)	0.006	0.012 (0.004)	0.006	0.012 (0.004)	0.006
Group (ref: control)			-0.057 (0.054)	0.288	-0.120 (0.057)	0.036			-0.119 (0.057)	0.038
Time (ref: post-intervention)			0.041 (0.018)	0.021	-0.018 (0.024)	0.450			-0.018 (0.024)	0.455
Group (ref: intervention X post-intervention)					0.123 (0.035)	<0.001			0.123 (0.036)	0.001
Situational awareness							-0.099 (0.053)	0.062	-0.002 (0.061)	0.975
Δ-2 restricted log-likelihood	783.662 (6)		787.379 (8)		779.571 (9)		784.207 (7)		783.340 (10)	
Residual	0.081 (0.004)	<0.001	0.081 (0.004)	<0.001	0.080 (0.004)	<0.001	0.081 (0.004)	<0.001	0.080 (0.004)	<0.001
Variance	0.114 (0.014)	<0.001	0.114 (0.014)	<0.001	0.114 (0.014)	<0.001	0.114 (0.014)	<0.001	0.114 (0.014)	<0.001

**Fig. 4:**
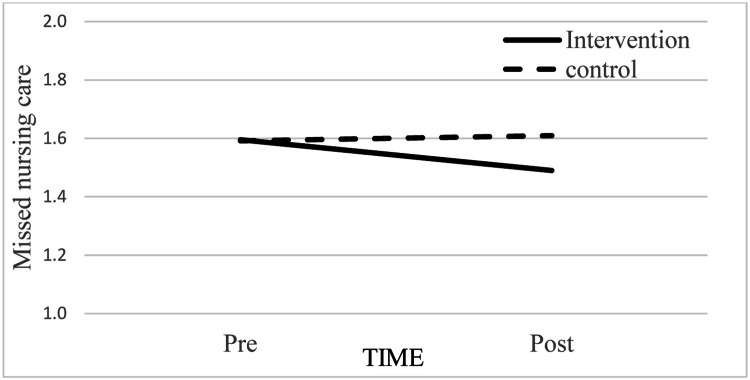
Effect of the interaction between time (pre vs. post) and group (intervention vs. control) on missed nursing care.

For missed nursing care (Table 3, Model 2), being a male (b =.245, p < .001), having less nursing experience (b =.005, p=.034), and increased workload (b =.011, p =.009) were all significantly associated with higher missed nursing care (Step 1). The direct effect of time on missed nursing care was also significant (b=.041, p=.021), whereas the direct effect of group was not (p > .05; Step 2). Yet, the interaction effect between time and group was significantly related to missed nursing care (Step 3; b = .123, p < .001). Closer inspection of Fig. 4 shows missed nursing care decreased from pre- to post-intervention in the intervention group, but no such effect was observed in the control group. These findings suggest that the huddle intervention effectively reduced missed nursing care.

Nevertheless, further analyses did not support the mediating role of situational awareness in the link between the huddle intervention and missed nursing care. Situational awareness was not significantly related to missed nursing care beyond the control variables (b= -.099, p =.062; Step 4). Additionally, when situational awareness was entered into the model beyond the effects of the intervention, it was not significant (b= -.002, p =.975; Step 5). These findings indicate that Hypothesis 2 was not supported.

**Fig. 5:**
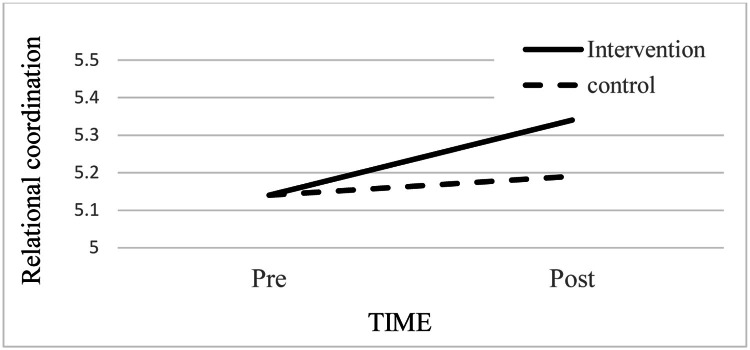
Effect of the interaction between time (pre vs. post) and group (intervention vs. control) on relational coordination.

Tables 4 presents the findings for the motivational mechanism, where relational coordination mediates the link between the group*time and missed nursing care. In Model 1, the control variables (seniority, gender, and workload) were not significantly associated with relational coordination (Step 1). However, the effect of time was significant (b = -.122, p < .001), indicating increased relational coordination post-intervention (Step 2), although the effect of group was non-significant (p > .05). The time and group interaction was significant (b = -.146, p < .035) (Step 3), showing that relational coordination was higher post-intervention in the intervention group compared to both pre-intervention and the control group (Fig. 5).

In Model 2, findings for missed nursing care replicated Steps 1-3 of Table 3, confirming that the interaction effect of time and group was significantly related to missed nursing care (Fig. 5). In Step 4, relational coordination was significantly associated with missed nursing care (b= -.131, p < .001). Furthermore, when relational coordination was entered in Step 5, it remained significant (b= -.125, p < .001), indicating that Hypothesis 3 was partially supported. While relational coordination improved performance and reduced missed nursing care, the direct interaction effect between time and group suggests that the intervention’s impact on missed nursing care was not fully mediated by relational coordination.

Table 4

**Table 4:** Mixed Linear Model - Motivational Mechanism.

	Model 1. Relational coordination as dependent variable					
Variables	Step1. Control variables		Step 2. Explanatory variables		Step 3. Interaction effect	
	Estimate (SE)	p-value	Estimate (SE)	p-value	Estimate (SE)	p-value
Experience as a nurse	-0.003 (0.005)	0.552	-0.003 (0.005)	0.505	-0.003 (0.005)	0.501
Gender (ref: female)	-0.196 (0.117)	0.096	-0.192 (0.117)	0.103	-0.193 (0.117)	0.100
Workload	0.004 (0.009)	0.679	-0.001 (0.009)	0.919	0.000 (0.009)	0.997
Group (ref: control)			0.076 (0.108)	0.481	0.150 (0.114)	0.190
Time (ref: post-intervention)			-0.122 (0.035)	<0.001	-0.053 (0.048)	0.272
Group (ref: control) X intervention (ref: post-intervention)					-0.146 (0.069)	0.035
Δ-2 restricted log-likelihood	2273.423 (6)		2268.226 (8)		2267.284 (9)	
Residual	0.325 (0.015)	<0.001	0.321 (0.015)	<0.001	0320 (0.015)	<0.001
Variance	0.461 (0.056)	<0.001	0.461 (0.056)	<0.001	0.462 (0.056)	<0.001

	Model 2. Missed nursing care as dependent variable									
Variable	Step 1. Control Variables		Step 2. Explanatory variables		Step 3. Interaction effect		Step 4. Mediation variable		Step 5. Mediation effect	
	Estimate (SE)	p-value	Estimate (SE)	p-value	Estimate (SE)	p-value	Estimate (SE)	p-value	Estimate (SE)	p-value
Experience as a nurse	0.005 (0.002)	0.043	0.005 (0.002)	0.034	0.005 (0.002)	0.033	0.005 (0.002)	0.052	0.005 (0.002)	0.041
Gender (ref: female)	0.245 (0.058)	<0.001	0.242 (0.058)	<0.001	0.243 (0.058)	<0.001	0.219 (0.056)	<0.001	0.219 (0.056)	<0.001
Workload	0.011 (0.004)	0.009	0.013 (0.004)	0.004	0.012 (0.004)	0.006	0.012 (0.004)	0.005	0.012 (0.004)	0.005
Group (ref: control)			-0.057 (0.054)	0.288	-0.120 (0.057)	0.036			-0.101 (0.055)	0.066
Time (ref: post-intervention)			0.041 (0.018)	0.021	-0.018 (0.024)	0.450			-0.025 (0.023)	0.289
Group (ref: intervention X post-intervention)					0.123 (0.035)	<0.001			0.105 (0.034)	0.002
relational coordination							-0.131 (0.015)	<0.001	-0.125 (0.015)	<0.001
Δ-2 restricted log-likelihood	783.662 (6)		787.379 (8)		779.571 (9)		714.027 (7)		716.576 (10)	
Residual	0.081 (0.004)	<0.001	0.081 (0.004)	<0.001	0.080 (0.004)	<0.001	0.076 (0.004)	<0.001	0.075 (0.004)	<0.001
Variance	0.114 (0.014)	<0.001	0.114 (0.014)	<0.001	0.114 (0.014)	<0.001	0.105 (0.013)	<0.001	0.105 (0.013)	<0.001

## 7 Discussion

This study introduces proactive huddles as a novel intervention aimed at reducing missed nursing care, offering a powerful tool to address this ongoing issue in healthcare, which continues to pose significant risks to patient safety and care quality (Jones et al., 2021). Beyond addressing the common structural improvements like increased staffing or enhanced teamwork (Schubert et al., 2021), this intervention explores two underlying mechanisms—cognitive and motivational—that mediate the relationship between proactive huddles and reduced missed nursing care. These mechanisms are crucial for understanding how the intervention operates within complex nursing environments, offering a deeper perspective on its efficacy.

Huddles represent a novel structural improvement in healthcare settings. While previous research has demonstrated their effectiveness in reducing safety incidents (Chapman et al., 2020; Pimentel et al., 2021; Murphy et al., 2023), the findings of the present study provide additional evidence that huddles may also help reduce missed nursing care. Our results align with those of Murphy et al. (2023), who found that in 57% of the huddles studied, issues related to missed tasks were identified and addressed (Burr et al., 2021; Guo et al., 2018; Lin et al., 2022). While the direct effects of huddles on reducing missed nursing care are clear, our study delves deeper into *how* this intervention achieves its impact on reduced missed nursing care through two key mechanisms: The motivational mechanism of relational coordination, that fosters teamwork and shared responsibility (Pimentel et al., 2021), and the cognitive mechanism of situational awareness that helps nurses better process and prioritize tasks (Ghaderi et al., 2022).

The motivational mechanism, which centered on relational coordination, demonstrated more promising results. As hypothesized, relational coordination, which emphasizes teamwork, shared goals, and effective communication, was found crucial in ensuring that essential nursing tasks are completed within the constraints of a dynamic and high-pressure environment (Havens et al., 2010). Apparently, the proactive huddles facilitated open discussions among nurses, enabling them to align their efforts, anticipate each other’s needs, and work collaboratively (Pimentel et al., 2021), eventually preventing missed care. The fact that relational coordination partially mediated the link between proactive huddles and missed nursing care supports previous studies’ conclusion regarding the central role of teamwork in ensuring that critical tasks are not delayed or omitted (Kalisch et al., 2013; Schubert et al., 2021). Namely, huddles probably fostered a culture of shared responsibility, which was then translated into reduced missed nursing care.

The cognitive mechanism addresses the role of situational awareness—a critical non-technical skill for nurses working in high-pressure environments (Endsley, 1995; Walshe et al., 2021). Situational awareness enables nurses to process complex information, make informed decisions, and anticipate patient needs by understanding the patient's clinical signs through both initial and ongoing evaluations, along with a comprehensive focus on the various dimensions of patient health (Ghaderi et al., 2022). Our findings, in line with previous research, showed improving situational awareness through huddles that provide structured opportunities for real-time information exchange, helping nurses process and prioritize tasks effectively (Chapman et al., 2020; Cosgrove et.al., 2023). While proactive huddles significantly enhanced situational awareness, our findings did not support its theoretical role as a mediator (Kanfer & Ackerman, 1989) between the intervention and reduced missed nursing care. First, the link between situational awareness and missed nursing care showed a trend, but it was not statistically significant. This may be explained by pre-intervention situational awareness levels within the sample, which (1) likely reflected systemic factors such as high workload, staffing shortages, and resource limitations that limited nurses’ ability to fully leverage or further develop their situational awareness; and (2) proactive huddles, at least as implemented in the current study, were not sufficient to significantly enhance situational awareness. Furthermore, even with improved situational awareness, the significantly increased volume of tasks and limited resources during the post-intervention period may have continued to overwhelm nurses, leading to care omissions despite their heightened awareness. These findings suggest that cognitive improvements alone may not be sufficient in environments with high workloads, although this mechanism remains a valuable component of the study’s innovation.

Together, this focus on both cognitive and motivational mechanisms is what sets this study apart from previous interventions. While earlier research has primarily looked at structural or process improvements, our study suggests that understanding the underlying mechanisms—how nurses cognitively process information and how they collaborate to deliver care—could offer more targeted strategies to reduce missed nursing care. These dual-mechanism approach suggests a new frontier in designing interventions, as they integrate both individual cognitive abilities and collective team dynamics to address a multifaceted problem.

### 7.1 Strengths and Limitations

This study has several notable strengths that contribute to the robustness of its findings. First, the randomized controlled design, with cluster randomization at the ward level, ensures that the observed differences between the intervention and control groups can be confidently attributed to the proactive huddles. This approach enhances the internal validity of the study (Steeger et al., 2021, particularly in a complex hospital setting. Moreover, the study captured data across multiple time points and shifts, allowing for a detailed understanding of how huddles impact nursing practices over time. The decision to collect data at the end of shifts minimized recall bias, ensuring that the nurses’ responses reflected recent experiences rather than distant memories (Te Braak et al., 2023).

However, there are several limitations to consider. First, the reliance on self-reported measures from nurses may introduce social desirability bias, as nurses could underreport missed care to avoid judgment. Nevertheless, prior research by Srulovici & Drach-Zahavy (2017) provides strong evidence supporting the validity of self-reports by showing a significant correlation between nurses' self-assessments and peer assessments, with no significant differences between the two. A second limitation is the lack of long-term follow-up, which prevents an assessment of whether the benefits of huddles are sustained over time. The findings therefore reflect only short-term effects of the intervention.

A further limitation concerns the possibility of participation and attrition bias, as some nurses declined due to workload, which may indicate underrepresentation of those under the greatest strain. However, overall agreement to participate was high (89%), with excellent completion rates (92% and 91%) and substantial engagement in huddles (84%). These figures are well within accepted standards for intervention studies, suggesting that while the risk of bias cannot be entirely excluded, it is unlikely to have materially affected the study’s conclusions. Finally, the exclusion of head nurses from the huddle process presents another limitation. Head nurses are key figures in ward management and decision-making (Pandaan et al., 2024), and their absence may have reduced the potential impact of the intervention on team coordination.

### 7.2 Recommendations for Further Research

Future research should include long-term follow-up to assess the sustainability of huddles’ effects and incorporate objective measures, such as direct observation or electronic health records, to complement self-reports. Examining the involvement of head nurses in huddles is also warranted, as their participation may strengthen relational coordination and enhance intervention outcomes.

Further investigation should focus on additional mechanisms through which huddles may reduce missed care. In particular, huddles may serve as brief micro-recovery pauses that help mitigate stress and cognitive overload (Albulescu et al., 2022; McGilton et al., 2023), provide a platform for enhancing nurses’ self-efficacy in prioritizing care (Pimentel et al., 2021), support informal communication, or facilitate situational prioritization strategies. It is also important to examine contextual conditions, including workload, staffing levels, and baseline cognitive abilities, to better understand why improvements *in situ*ational awareness may not always translate into reduced missed care.

Lastly, future studies should explore moderating and mediating factors that influence the effectiveness of huddles, such as safety culture. Identifying these influences will help clarify the conditions under which huddles are most effective, particularly in high-stress environments.

## 8 Conclusion

This study demonstrates the effectiveness of proactive huddles as a cost-effective and scalable intervention to reduce missed nursing care, providing structured platforms for real-time communication and team coordination. By testing two key mechanisms—situational awareness and relational coordination—the study advances our understanding of how huddles impact care delivery, moving beyond traditional structural interventions. The motivational mechanism of relational coordination emerged as a critical driver of reduced missed nursing care, reinforcing the importance of team-based approaches. In comparison, the findings did not support the cognitive mechanism. While situational awareness improved in response to the intervention, it was not sufficient to decrease missed nursing care, and did not mediate the intervention- missed nursing care link. Perhaps, the increased workload may have limited its full effect, highlighting the complexity of missed care in high-stress environments. These findings suggest that addressing both cognitive processes and team dynamics is essential to improving care quality. By integrating both cognitive and motivational processes, this dual-mechanism approach offers a new framework for interventions in high-stress healthcare environments such as intensive care units or emergency departments, where missed care poses a critical risk. Importantly, because omissions in care directly compromise patient safety (Papathanasiou et al., 2024), our findings add to the broader literature demonstrating that huddles are not only effective in improving teamwork and coordination but may also play a vital role in enhancing the safety and reliability of care delivery. In this vein, while our huddle script already included prompts addressing safety and task completion, future implementations could be strengthened by explicitly asking about patients at risk for deterioration, anticipated safety concerns during the shift, and the availability of essential equipment. Such refinements may further enhance huddles’ role in fostering both completeness of care and a culture of safety vigilance.

## Funding statement

This study was supported by a grant from the Cheryl Spencer Institute of Nursing Research, The University of Haifa, Israel. The first author, a Ph.D. candidate, received a scholarship that allowed her to dedicate time to conducting the study.

## CRediT authorship contribution statement

**Marina Vexler:** Writing – original draft, Visualization, Validation, Software, Resources, Project administration, Methodology, Investigation, Formal analysis, Data curation, Conceptualization. **Anat Drach-Zahavy:** Writing – review & editing, Writing – original draft, Visualization, Validation, Supervision, Software, Resources, Project administration, Methodology, Investigation, Funding acquisition, Formal analysis, Data curation, Conceptualization. **Einav Srulovici:** Writing – review & editing, Writing – original draft, Visualization, Validation, Supervision, Software, Resources, Project administration, Methodology, Investigation, Funding acquisition, Formal analysis, Data curation, Conceptualization.

## Declaration of competing interest

The authors declare the following financial interests/personal relationships which may be considered as potential competing interests:

Marina Vexler reports financial support was provided by University of Haifa The Cheryl Spencer Institute for Nursing Research. Marina Vexler reports a relationship with University of Haifa The Cheryl Spencer Institute for Nursing Research that includes: funding grants. If there are other authors, they declare that they have no known competing financial interests or personal relationships that could have appeared to influence the work reported in this paper.

## Appendix Supplementary materials

Image, application 1

Image, application 2
