# Supplementary material for: Proactive huddles to reduce missed nursing care; the mediating roles of personal situational awareness and rational coordination: A cluster randomized pre post intervention study
Source: Int J Nurs Stud Adv. 2025 Nov 10;9:100448. doi: 10.1016/j.ijnsa.2025.100448 (PMC12670451; doi:10.1016/j.ijnsa.2025.100448)
Supplement: Supplementary file 1 [file mmc1.docx]

Supplementary Material 1 **Form of Huddles**

**Form of Huddles**

(to be filled by a huddle leader)

|  | date |  | ward |
| --- | --- | --- | --- |
|  | Beginning time |  |  |
|  | End time |  | Total nurses on shift |
|  | leading a meeting |  | number of participating in the meeting |

**Leading questions:**

What are the two main things that delay nursing care in the current shift?

Are there any activities that you don't think will be enough by the end of the shift?

What prevents or what is missing to complete these actions?

Do you need help?

How do you think we can take care of these gaps?

| **subject** |  |  |
| --- | --- | --- |
| Were there any problems/gaps during the Huddles? | Yes/No | Detail_____________  Detail_____________ |
| Are there solutions to the problems/gaps that have been flooded? | Yes/No | Detail_____________  Detail_____________ |
| Were there any unusual events during the shift (resuscitation, fall in hospital, incident with family members or other) | Yes/No | Detail_____________  Detail_____________ |
| Describe the atmosphere during the Huddles (positive, burden, attentive and cooperative people, disturbances, etc.) |  |  |
| Were decisions made and/or tasks defined during a Huddles? | Yes/No |  |
| decisions/tasks | who is responsible | |
|  |  | |
|  |  | |
