# Supplementary material for: Proactive huddles to reduce missed nursing care; the mediating roles of personal situational awareness and rational coordination: A cluster randomized pre post intervention study
Source: Int J Nurs Stud Adv. 2025 Nov 10;9:100448. doi: 10.1016/j.ijnsa.2025.100448 (PMC12670451; doi:10.1016/j.ijnsa.2025.100448)
Supplement: Supplementary file 2 [file mmc2.docx]

Supplementary Material 2 **The huddle script**

**Topics for Huddles**

**Ensure that the tasks you are responsible for are completed**

**Are all the tasks completed?**

| Administering medication | | Patient downloads/Patient Mobility | Bathing |
| --- | --- | --- | --- |
| Admission of a new patient | Repeated assessments | Wound care | Assessments |
| Preparing a patient for surgery/test | | Patient discharge | Patient Education |
| Proactive conversation | Emotional support for the patient and his family | Registering and reporting on activities performed | Receiving a patient from surgery/test |

דדשד

**Share your experience**

Tell about a case when a huddle helped you complete a nursing task

**Identify the gaps**

| Who doesn't finish the tasks by the end of the shift? | Who needs help |
| --- | --- |
|  | Missing equipment |
